# Comparative transcriptome analysis reveals potential evolutionary differences in adaptation of temperature and body shape among four Percidae species

**DOI:** 10.1371/journal.pone.0215933

**Published:** 2019-05-07

**Authors:** Peng Xie, Shao-Kui Yi, Hong Yao, Wei Chi, Yan Guo, Xu-Fa Ma, Han-Ping Wang

**Affiliations:** 1 Aquatic Genetics and Breeding Laboratory, The Ohio State University South Centers, Piketon, OH, United States of America; 2 College of Fisheries, Huazhong Agricultural University, Wuhan, Hubei, China; 3 Fisheries Research Institute of Xinjiang Uygur Autonomous Region, Urumqi, Xinjiang, China; Natural History Museum of London, UNITED KINGDOM

## Abstract

Considering the divergent temperature habitats and morphological traits of four Percidae species: yellow perch (*Perca flavescens*), Eurasian perch (*Perca fluviatilis*), pike perch (*Sander lucioperca*), and ruffe (*Gymnocephalus cernua*), we stepped into the transcriptome level to discover genes and mechanisms that drive adaptation to different temperature environments and evolution in body shape. Based on 93,566 to 181,246 annotated unigenes of the four species, we identified 1,117 one-to-one orthologous genes and subsequently constructed the phylogenetic trees that are consistent with previous studies. Together with the tree, the ratios of nonsynonymous to synonymous substitutions presented decreased evolutionary rates from the *D*. *rerio* branch to the sub-branch clustered by *P*. *flavescens* and *P*. *fluviatilis*. The specific 93 fast-evolving genes and 57 positively selected genes in *P*. *flavescens*, compared with 22 shared fast-evolving genes among *P*. *fluviatilis*, *G*. *cernua*, and *S*. *lucioperca*, showed an intrinsic foundation that ensure its adaptation to the warmer Great Lakes and farther south, especially in functional terms like “Cul4-RING E3 ubiquitin ligase complex.” Meanwhile, the specific 78 fast-evolving genes and 41 positively selected genes in *S*. *lucioperca* drew a clear picture of how it evolved to a large and elongated body with camera-type eyes and muscle strength so that it could occupy the highest position in the food web. Overall, our results uncover genetic basis that support evolutionary adaptation of temperature and body shape in four Percid species, and could furthermore assist studies on environmental adaptation in fishes.

## Introduction

While reading the book *Adaptation and Natural Selection*, the preface sentence “Natural selection is the only acceptable explanation for the genesis and maintenance of adaptation,” will certainly resonate with ecologists [1]. Interestingly, “adaptive evolution” and “evolutionary adaptation” have been documented by researchers’ studies on numerous organisms; either way, natural selection is still the most critical part. Undoubtedly, both adaptation and evolution are inseparable from organisms and the environment. Organisms need to adapt to various environments, and in turn, specific environments drive the evolution of organisms. One of the fundamental concerns in molecular evolution often focuses on the role of adaptation, for instance, the relationship between adaptive evolution and neutral evolution [2]. In the process of evolution, the stresses that a species adapts to an environment often lead to adjustments of key genes, as well as changes in traits [3–6]—this is the power of selection. Thus, one of the central interests is to discover potential genes and mechanisms that are subject to such power. With advances in next-generation sequencing techniques and bioinformatic analyses, the ratio, numbers, and patterns of nonsynonymous and synonymous substitutions in protein-coding genes could be computed and applied to detect selection [7–11]. To be noted, RNA-seq is one of the frequently applied and efficient methods to reveal information like transcriptomes and expression of genes, even without genome annotation [12–14].

Fish, like most vertebrates, present a diverse range of divergent evolutions and global environmental adaptations. As ectotherms, fish are very sensitive to ambient temperature [15, 16], as well as other abiotic factors; and temperature powerfully influences biological functions like embryo development, metabolic rate, and growth [17–20]. These functions and genes involved usually contribute to the formation of traits [21, 22]. Most functions and gene expression are regulated by environmental factors like temperature, dissolved oxygen, and osmotic pressure. Thus, influences like this often determine the fitness of fish in diverse environments. For instance, the body could be well shaped for better swimming and occupying a unique ecological niche. Such fitness should be the purposeful evolution of fish during their adaptation, and may eventually lead to the speciation. For example, in Lake Stechlin, the vertical difference in water temperature drove temperature-related physiological adaptations that promote ecological evolution in the sympatric species pair of *Coregonus* spp [23]. The divergence in ontogenetic rates found in a Nordic freshwater fish, presenting in differentiation of larval developmental rate and efficiency, was also driven by temperature and proved that the power of natural selection could promote the evolutionary adaptation of temperate lake fish within only 22 generations [24]. In Lake Victoria, divergent environmental selections drove divergency in sensory systems and ultimately led to the speciation of two cichlid fishes [25]. The three-spine stickleback (*Gasterosteus aculeatus*), a star model for adaptive evolution research and whose ancestor originated from marine environment, showed typical evolutionary changes in its global distribution and colonization in freshwater [26–28], especially in kidney morphology and candidate gene expression [29]. Moreover, in cichlid fishes, sticklebacks, salmons, and *Gnathopogon* fishes, genomic architectures contained the basic habitat-related mechanisms of adaptive evolution for ecologically divergent body shape in sympatric species [30–33].

Here, we turned attention to four Percidae species: yellow perch (*Perca flavescens*), Eurasian perch (*Perca fluviatilis*), pike perch (*Sander lucioperca*), and ruffe (*Gymnocephalus cernua*). We were interested in the two following points:

First, whether there exist intrinsic molecular differences that explain the divergent adaptation of the two sister species *P*. *flavescens and P*. *fluviatilis* to different temperature environments. Referring to the hypotheses on the origin of percids, especially the Laurasian origin hypothesis, *P*. *flavescens* originated from the same place as the three sympatric species of *P*. *fluviatilis*, *G*. *cernua*, and *S*. *lucioperca*, but had adapted to the warmer North American Great Lakes and the farther south as a result of the vicariant speciation of North America and Europe due to the opening of the North Atlantic [34–37]. However, *P*. *fluviatilis* is mainly distributed in the colder north region of Eurasia, especially in the Irtysh River basin. Therefore, natural selection seems to have more influence on evolution in the warm acclimation of *P*. *flavescens*.

Second, what about the evolutionary differences that support the adaptation in body shape? Despite a long term of adaptive evolution, *P*. *flavescens* and *P*. *fluviatilis* are still very similar in biology [38]. They share a small and high body with *G*. *cernua*, while *S*. *lucioperca* maintains a larger and elongated body. Moreover, during our investigation of the fishery resources in the Irtysh River in China, we found that *S*. *lucioperca* preyed on *P*. *fluviatilis* and *G*. *cernua*, including some other small fishes. It is likely that *S*. *lucioperca* had evolved into, and occupied, a higher position of the food web inside the ecosystem of the Irtysh River than did the other two sympatric species. In this study, we conducted comprehensive investigations through RNA-sequencing of the four species and bioinformatic analysis to explore the above assumptions and evolutionary mechanisms that support ecological adaptation of temperature and body shape among these four Percid species.

## Materials and methods

### Ethic statement

We confirmed that all the methods and experimental protocols of this study were performed in accordance with guidelines and regulations approved by the animal ethics committee of The Ohio State University and the *Guidelines for Experimental Animals* of the Ministry of Science and Technology (Beijing, China; No. [2006]398, 30 September 2006). We minimized the suffering on individuals and influence on natural resources.

### Collection of temperature and morphological data

**Fig 1:**
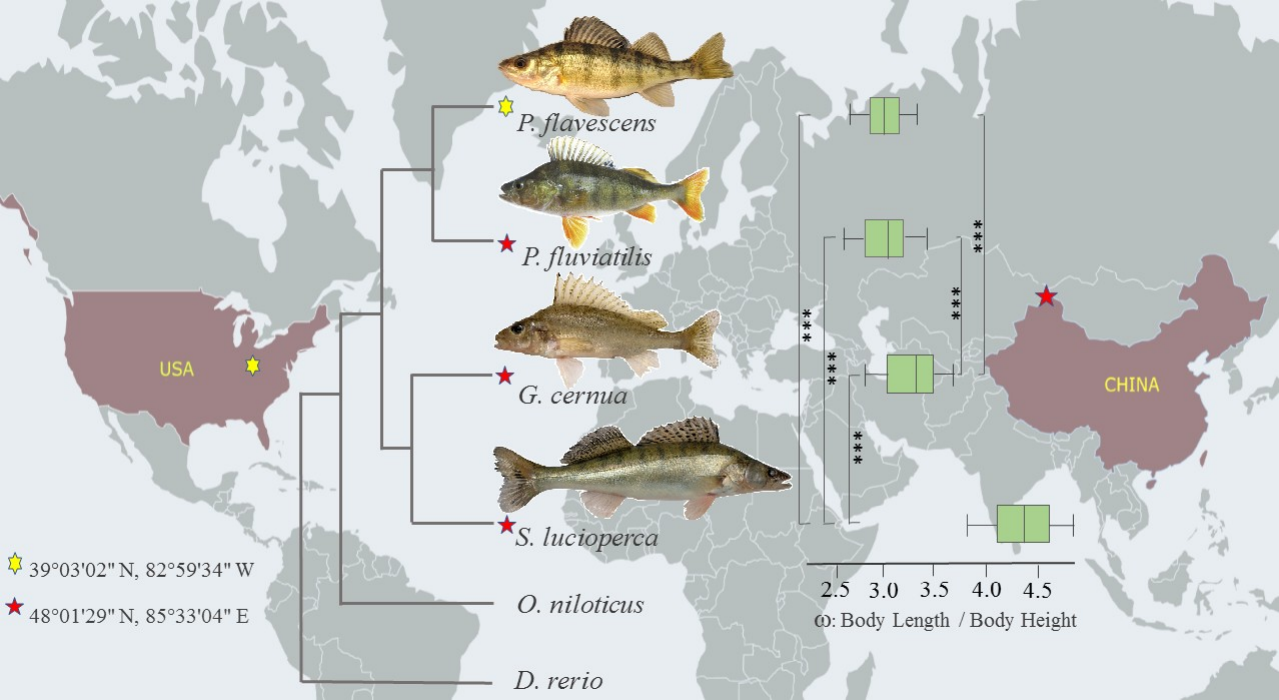
Sampling location, phylogenetic tree, and multiple comparison of ratios of body length / body height (ω) among four Percids. ***: significant difference (*p* < 0.01) revealed from nonparametric multiple comparisons (npmc) using the “npar” package in R 3.5.3.

With the accessible data in CoastWatch (https://coastwatch.glerl.noaa.gov/statistic/statistic.html), we summarized the average annual water temperature of Lake Erie from 1992 to 2017 as a reference. We collected *P*. *flavescens* from the first generation of wild population from ponds in Piketon, Ohio, USA (39°03'02" N, 82°59'34" W, 380 km south of Lake Erie center). The parents of *P*. *flavescens* were introduced from the Perquimans River, North Carolina, USA (36°11'38" N, 76°27'36" W) in 2010. We had also recorded the water temperature of the ponds for the past several years. We collected the individuals of *P*. *fluviatilis*, *G*. *cernua*, and *S*. *lucioperca* in the Irtysh River, Xinjiang Uygur Autonomous Region, northwest of China (48°01'29" N, 85°33'04" E) (Fig 1). The Irtysh River and Ob River, chief upper streams of the Ob-Irtysh River, both arise from their source in the glaciers of the Altai mountains, south and north side respectively, while they flow almost parallel through most of the cold flat Siberian plains and eventually converge and inject into the Arctic Ocean. Due to the absence of temperature data for the Irtysh River in the past decades, we randomly monitored the data from early May to early October from 2012 to 2016. In addition, we also referred the climate temperature data from the World Climate website (https://www.climate-charts.com/) for the two sampling locations.

We collected the data of body length (BL), body height (BH), and body weight as proxies for growth of the four Percids. In addition, we compared the ratios of BL/BH, directly reflecting the body shape of fish [39, 40], among the four Percids. We conducted the non-parametric multiple comparison of the ratios using the “npar” package in R 3.5.3 because the sample sizes and variance of the ratios were not equal [41]. Furthermore, we also computed the asymptotic length *L*_∞_ and the growth performance index ∅ between *P*. *fluviatilis* and *S*. *lucioperca* based on the data of age and body length since the *L*_∞_ and ∅ could statistically reveal the growth characteristic of fish [42, 43]. We finally collected two datasets of length and age from 386 individuals for *P*. *fluviatilis* and 176 for *S*. *lucioperca*, which matched the requirements of sample size described in a previous study [44]. We estimated the *L*_∞_ through the Von Bertalanffy growth function (VBGF) with Levenberg-Marquardt’s nonlinear fitting in Origin 2018 (Originlab, Northampton, USA) [45]. The VBGF and formula for calculating ∅ were as follows:

$$L_{t}=L_{\infty }[1-e^{-k(t-t_{0})}],\;\emptyset =\log_{10}k+2\;\log_{10}L_{\infty },$$

where *L_t_* is the body length at age of *t*; *k* and *t*_*0*_ denote growth coefficient and scaling constant, generated by the nonlinear fitting, respectively.

### Sample collection and RNA extraction

We collected tissue samples of brain, heart, skin, dorsal muscle, liver, spleen, vertebra, and fins from three individuals of each species for RNA extraction in the summer of 2015. We snap-froze samples of *P*. *flavescens* in liquid nitrogen to a temperature of -80°C, while samples of *P*. *fluviatilis*, *G*. *cernua*, and *S*. *lucioperca* stored in ice cooling TRIzol were brought to a temperature of -80°C according to field conditions. We isolated total RNA of the samples using an improved protocol for better next-generation sequencing [46]. We checked the purity of RNA using the NanoPhotometer spectrophotometer (IMPLEN, CA, USA), and measured the concentration using Qubit RNA Assay Kit in Qubit 2.0 Fluorometer (Life Technologies, CA, USA). We assessed the integrity using the RNA Nano 6000 Assay Kit of the Agilent Bioanalyzer 2100 system (Agilent Technologies, CA, USA). Finally, we prepared equal amount of 3 μg RNA for each specimen.

### Sequencing, assembly, and annotation

We purified the mRNA from total RNA using poly-T oligo-attached magnetic beads and subsequently fragmented them with fragmentation buffer. We synthesized first-strand cDNAs with random hexamer primer and generated pair-strands cDNAs, followed by purification using AMPure XP beads. Then we sequenced the qualified cDNA libraries on an Illumina Hiseq 2500 platform and obtained paired-end reads. We firstly processed raw data (raw reads) of fastq format within FastQC Version 0.11.4 [47] by removing reads containing adapter, ploy-N, and low quality reads from the raw data. We conducted transcriptome assembly using Trinity with default sets [13]. Redundant unigenes were removed through CD-HIT-EST program with parameters “c = 0.95” and “n = 10” [48]. Then, we annotated function of unigenes using BLASTX (E-value of 1 e-5) based on the following databases: Nr (NCBI non-redundant protein sequences), Nt (NCBI non-redundant nucleotide sequences), Pfam (Protein family), KOG/COG (Clusters of Orthologous Groups of proteins), Swiss-Prot (A manually annotated and reviewed protein sequence database), KEGG (KEGG Ortholog database), and GO (Gene Ontology), after which the best aligning results were used for the direction of sequences.

### Identification of orthologous genes

To identify orthologous genes, we downloaded annotated CDSs (coding sequences) and proteins of one Perciformes species, *Oreochromis niloticus*, and one Cypriniformes species, *Danio rerio*, from the Ensembl database as references. Then we filtered all the putative proteins of the four Percidae species, together with two downloaded proteomes datasets, to build the local database for blasting parameters so that length of proteins must be longer than 50 and percentage of stop codon must be less than 20. Subsequently, we conducted self-to-self BLASTP for all amino acid sequences with a cut-off E-value of 1e-5 in the local database. We constructed orthologous groups from the BLASTP results with OrthoMCL v2.0.3 using default settings [49]. We retained the longest unigene if multiple unigenes were orthologous. We aligned all the orthologous genes in groups that have one-to-one relationships among lineages by PRANK [50] and subsequently trimmed them by Gblocks [51] with the parameter “-codon” and “-t = c,” respectively.

### Phylogenetic analysis and substitution rate estimation

We concatenated the trimmed sequences of orthologous genes into super-alignments in FasParser2 [52] for phylogenetic analysis. We constructed the Bayesian tree based on the concatenation of all one-to-one orthologous genes in MrBayes 3.2.6 [53] using the “GTR+I” model selected by AIC in MrModeltest 2.4 [54], and inferred the ML tree in PAUP [55].

Basing on the consensus phylogenetic tree, we estimated the substitution rates using the codeml program in FasParser2. To be specific, 1) we used the free ratio model (model = 1, NSsites = 0) to estimate the evolutionary rates of every lineage at each of the 1,117 orthologous genes and the super-alignments (ratios of the alignments were regarded as references only), filtering out the genes if N × d_N_ or S × d_S_ < 1 or d_S_ > 1 [56], and 2) to identify genes that might contribute to lineage-specific adaptations, we conducted the branch model (model = 2, NSsites = 0) and branch-site model (model = 2, NSsites = 2) to explore the following two evolutionary gene sets for the four Percidae fishes: fast-evolving genes (FEGs) that have undergone higher d_N_/d_S_ ratios (the ratio of nonsynonymous to synonymous substitutions) in specific lineages in comparison with the rest branches, and positively selected genes (PSGs) (1 < d_N_/d_S_ < 999) that contain specific codon sites influenced by positive selection in each foreground branch only. All the results were automatically filtered by FasParser2 according to default settings, including likelihood ratios tests (LRTs) among alternative models, false discovery rate (FDR) correction, and Bayes Empirical Bayes (BEB) estimation. Then, to figure out potential functions of both FEGs and PSGs, we performed gene ontology (GO) functional enrichment analysis in DAVID (https://david.ncifcrf.gov/tools.jsp) [57].

## Result

### Differences in environmental temperature

The mean annual water temperature (*T*_*mw*_) of ponds at OSU Aquaculture Research Center at Piketon was 18.4°C from 2014 to 2016 (Table 1), compared to 11.3°C in Lake Erie (S1 Table in supplemental file). The data of water temperature we randomly monitored in the Irtysh River revealed a comparatively low mean of 11.2°C during the warmer seasons, late spring to middle autumn (S2 Table). However, recorded data showed that mean temperatures in January ranged from -28°C on the shores of the Kara Sea to -16°C in the upper reaches of the Irtysh River, and July temperatures for the same locations, respectively, ranged from 4°C to merely 20°C [58]. Notably, the absolute minimum temperature in the Altai Mountains could be as extremely low as -60°C, and this area has short warm summers and long cold winters, i.e., the freezing period of the Irtysh River usually lasts from late November to late March with a mean water temperature of 0°C to 3°C [59]. We could conclude that the mean water temperature in the Irtysh River should be lower than 11.2°C.

**Table 1:** Temperature and morphological information about four Percids.

	range of *T*_*c*_ (°C)	*T*_*mw*_ (°C)	*L*_∞_ (mm)	k	range of ω	mean of ω	∅
*P*. *flavescens*	-6.5 ~ 30.2	18.4	N.A.	N.A.	2.83 ~ 3.28	3.03	N.A.
*P*. *fluviatilis*	-21.8 ~ 28.2	< 11.2	498.48	0.17	2.66 ~ 3.52	3.04	4.63
*G*. *Cernua*	-21.8 ~ 28.2	< 11.2	N.A.	N.A.	2.91 ~ 3.61	3.23	N.A.
*S*. *lucioperca*	-21.8 ~ 28.2	< 11.2	1091.11	0.09	3.74 ~ 4.83	4.30	5.05

*T*_*c*_ = mean values of monthly low and high climate temperature, data from World Climate; *T*_*mw*_ = mean annual water temperature of habitats; *L*_∞_ = asymptotic length; k = growth coefficient; ω = ratio of body length / body height; ∅ = growth performance index; N.A. = data were not available here.

Thus, it is obvious that the Great Lakes and farther south are much warmer than the Irtysh River basin. Moreover, the three sympatric species are enduring a wider fluctuation of temperature than *P*. *flavescens* does, 60.0°C and 36.7°C, respectively.

### Differences in morphological traits among four species

The asymptotic length of *S*. *lucioperca* was 1,091.11 mm, which was two times longer than 498.48 mm for *P*. *fluviatilis*. The ratios of body length / body height showed that the body of *S*. *lucioperca* was more elongated with the largest mean ratio of 4.30, ranging from 3.74 to 4.83, followed by *G*. *Cernua*, *P*. *fluviatilis*, and *P*. *flavescens*, with the mean ratios of 3.23, 3.04, and 3.03, respectively (Table 1 and Fig 1, S3 Table). Moreover, the growth performance index of *S*. *lucioperca* (∅ = 5.05) was higher than *P*. *fluviatilis* (∅ = 4.63) (Table 1).

### Transcriptome assembly and annotation

After the quality control and trinity assembly described above, we got 129,971 to 325,637 transcripts for the four Percidae fish. Subsequently, we obtained 181,246, 93,566, 102,696, and 128,467 unigenes for *P*. *flavescens*, *P*. *fluviatilis*, *G*. *Cernua*, and *S*. *lucioperca*, respectively (Table 2). The unigenes were multiply annotated against major protein databases (e.g. NR, GO, KEGG, and Swiss-Prot).

**Table 2:** Basic assemble information of transcriptomes for four Percids.

	*N*_*c*_	*N*_*t*_	*N*_*u*_	mean length	median length	N50	GC%
*P*. *flavescens*	20,271,970	325,637	181,246	746	472	929	44.68%
*P*. *fluviatilis*	62,944,950	129,971	93,566	699	360	1,223	48.17%
*G*. *Cernua*	30,778,283	158,925	102,696	705	345	1,296	45.73%
*S*. *lucioperca*	65,147,286	186,976	128,467	610	326	955	47.20%

*N*_*c*_ *=* number of clean reads, *Nt =* number of transcripts, *N*_*u*_ = number of unigenes, N50 = minimum contig length needed to cover 50% of assembled transcriptome

### Identified orthologous groups and phylogenetic relationship

We identified a total of 1,117 one-to-one orthologous genes, the lengths of which ranged from 150 to 3,504 bp, and subsequently concatenated them for phylogenetic analysis. The phylogenetic trees constructed in different software were consensus and consistent with the topology of previous researches [60, 61], that is, the two sister species *P*. *flavescens* and *P*. *fluviatilis* were in the terminal clade and subsequently converged with the branch clustered by *G*. *Cernua* and *S*. *lucioperca* (Fig 1).

### Evolutionary rates in the Percidae lineages

**Fig 2:**
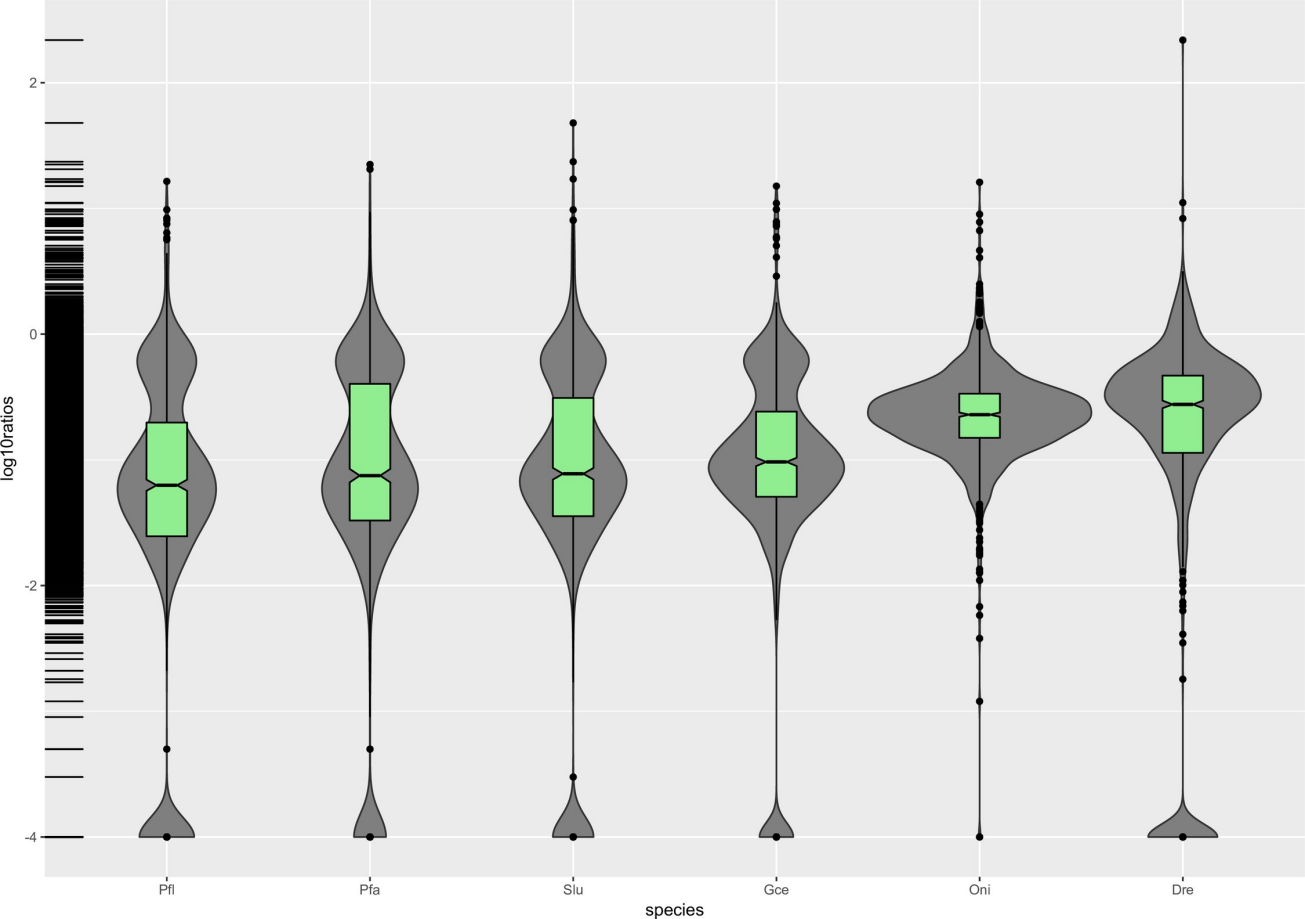
Violin plot of d_N_/d_S_ ratios for terminal branches. Pfl: *P*. *fluviatilis*, Pfa: *P*. *flavescens*, Slu: *S*. *lucioperca*, Gce: *G*. *cernua*, Oni: *O*. *niloticus*, Dre: *D*. *rerio*; log10ratios: to better remain all the 1027 values and display the tendency, the ratios were log-transformed and plotted in R 3.5.3.

During the evolution of species, especially within lineages, the selective pressure is presented as the differences of d_N_/d_S_ ratio at the gene level [9]. After filtering out outliers, the averages of d_N_/d_S_ ratios revealed from 1,027 orthologues displayed decreasing trends from the *D*. *rerio* branch to the sub-branch clustered by *P*. *flavescens* and *P*. *fluviatilis*, with the highest value of 0.587 for *D*. *rerio* and lower values of 0.310 and 0.283 for *P*. *flavescens* and *P*. *fluviatilis*, respectively (S4 Table, Fig 2). Meanwhile, the d_N_/d_S_ ratios revealed from concatenated alignments of the 1,117 orthologues also indicated the same trends. However, we just regarded these ratios and trends as references and did not use them for subsequent analyses, in case of unreliable high d_N_/d_S_ produced by errors in the alignments. For the five Perch-likes lineages, 36 genes of higher evolutionary rate were identified in the *P*. *fluviatilis* lineage followed by 42, 41, 47, and 91 in *P*. *flavescens*, *G*. *cernua*, *S*. *lucioperca*, and *O*. *niloticus*, respectively (141 in *D*. *rerio*).

### Fast-evolving and positively selected genes

In total, we identified 247, 210, 249, and 237 FEGs for *P*. *flavescens*, *P*. *fluviatilis*, *G*. *cernua*, and *S*. *lucioperca*, respectively. Whilst, we identified 64, 47, 58, and 48 PSGs, respectively. Furthermore, we also counted the subset of overlapping genes after comparison of the two sets above, while such genes were considered to be directly related to adaptive evolution [7, 62]. Finally, we identified 23, 14, 25, and 6 overlapping genes in *P*. *flavescens*, *P*. *fluviatilis*, *G*. *cernua*, and *S*. *lucioperca*, respectively (Table 3).

**Table 3:** Average d_N_/d_S_ values, the number of FEGs, PSGs and overlapping genes between FEGs and PSGs for the four Percids.

Species	d_N_/d_S_	FEGs	PSGs	Overlapping
*P*. *flavescens*	0.310	247	64	23
*P*. *fluviatilis*	0.283	210	47	14
*G*. *cernua*	0.306	249	58	25
*S*. *lucioperca*	0.367	237	48	6

d_N_/d_S_: average ratios of nonsynonymous to synonymous substitutions; FEGs: fast-evolving genes; PSGs: positively selected genes

Notably, the 60 overlapping genes were significantly (p < 0.05) enriched in only two “biological process” (BP) terms (tRNA aminoacylation, and regulation of vascular endothelial growth factor receptor signaling pathway) and three KEGG pathways (dre00630: glyoxylate and dicarboxylate metabolism, dre01100: metabolic pathways, dre01200: carbon metabolism).

### Functional annotation of shared and specific FEGs / PSGs

Among the three cold-sympatric species, there were 22 shared FEGs that were significantly enriched in only one BP term (translational initiation). In addition to the two genes enriched, we manually annotated the rest of the 20 genes against the UniProt database. Meaningful terms like “ion binding,” “protein transport,” and “ubiquitin-dependent protein catabolic process” were assigned and reported to be closely related to cold stress [63].

In the *P*. *flavescens* lineage, there were 93 specific FEGs and 57 specific PSGs. These 93 specific FEGs were significantly assigned to four BP terms, three “cell component” (CC) terms, three “molecular function” (MF) terms, and one KEGG pathway. The 57 specific PSGs were significantly assigned to five BP terms, four CC terms, one MF term, and one KEGG pathway (Table 4). Among these GO terms and pathways enriched form the two gene sets, notably, the “endoplasmic reticulum membrane” (GO: 0005789) and “cytoplasm” (GO: 0005737) were significant in both sets.

**Table 4:** Significantly enriched terms among FEGs and PSGs for *P*. *flavescens*.

	category	term	GO number	genes involved	*p* value
FEGs	biological process	transcription initiation from RNA polymerase II promoter	GO:0006367	*crsp7*, *gtf2a2*, *ercc3*	0.002
		protein processing	GO:0016485	*ncstn*, *pcsk2*, *aph1b*	0.014
		oxidation-reduction process	GO:0055114	*hao*,*1 sdha*, *uevld*, *cyp1a*, *hif1an creg*,*2 dhtkd1*, *cox15*	0.016
		translation	GO:0006412	*eif3i*, *iars*, *mrpl13*, *mrpl15*, *mrpl47*	0.046
	cellular component	holo TFIIH complex	GO:0005675	*gtf2h4*, *ercc3*	0.021
		endoplasmic reticulum membrane	GO:0005789	*cyp1a*, *ttc9b*, *sdf2l1*, *fkbp3*, *pigb*	0.026
		cytoplasm	GO:0005737	*nit2*, *stk10*, *hal*, *vbp1*, *ufc1*, *pin4*, *naa38*, *plaa*, *iars*, *eif4a3*, *psmg1 hif1an*, *cnep1r1*, *tmem216*, *grcc10*, *eif3i*, *phlda3*	0.026
	molecular function	peptidyl-prolyl cis-trans isomerase activity	GO:0003755	*ttcpb*, *ppil1*, *fkbp3*, *pin4*	0.0006
		oxidoreductase activity	GO:0016491	*hao1*, *sdha*, *uevld*, *cyp1a*, *hif1an*, *creg2*, *dhtkd1*, *hpgd*, *bdh1*	0.001
		RNA polymerase II carboxy-terminal domain kinase activity	GO:0008353	*gtf2h4*, *ercc3*	0.019
	KEGG pathway	Basal transcription factors	dre03022	*gtf2a2*, *gtf2h4*, *ercc3*	0.019
PSGs	biological process	SCF-dependent proteasomal ubiquitin-dependent protein catabolic process	GO:0031146	*rnf7*, *fbxw5*, *fbxl5*	0.0004
		protein ubiquitination	GO:0016567	*fbxw5*, *vhl*, *fbxl5*, *fbxo045*, *rnf170*	0.001
		phenylalanyl-tRNA aminoacylation	GO:0006432	*farsb*, *lrrc47*	0.011
		odontogenesis	GO:0042476	*cyp26b1*, *ext2*	0.029
		regulation of vascular endothelial growth factor receptor signaling pathway	GO:0030947	*hif1an*, *vhl*	0.029
	cellular component	endoplasmic reticulum membrane	GO:0005789	*ttc9b*, *cyp26b1*, *pigb*, *alg5*, *rnf170*	0.004
		phenylalanine-tRNA ligase complex	GO:0009328	*farsb*, *lrrc47*	0.007
		cytoplasm	GO:0005737	*taldo1*, *fbxw5*, *hif1an*, *psme2*, *parsb*, *fbxl5*, *ufc1*, *grcc10*, *pbdc1*, *pin4*, *tradd*, *tnp03*	0.016
		Cul4-RING E3 ubiquitin ligase complex	GO:0080008	*rnf7*, *fbxw5*	0.021
	molecular function	phenylalanine-tRNA ligase activity	GO:0004826	*farsb*, *lrrc47*	0.010
	KEGG pathway	Metabolic pathways	dre01100	*prim1*, *fahd1*, *ndufb5*, *taldo1*, *cyp26b1*, *pigb*, *alg5*, *mgll*, *ext2*, *adpgk2*	0.007

In the *S*. *lucioperca* lineage, the 78 specific FEGs were significantly assigned to five BP terms, one CC term, two MF terms, and five KEGG pathways. The 41 specific PSGs were significantly enriched in one BP term and two CC terms (Table 5). Among the terms above, the “proteolysis” and “proteolysis involved in cellular protein catabolic process” play beneficial roles in skeletal muscle growth and stress adaptation during the long-term viability and maintenance of any organ system [64]. The “phospholipid biosynthetic process” was positively stimulated by growth factors like TGF-β1, IGF-1, and BMP-2, as seen in a previous study on the fibroblast-like synoviocytes [65].

**Table 5:** Significantly enriched terms among FEGs and PSGs for *S*. *lucioperca*.

	category	term	GO number	genes involved	*p* value
FEGs	biological process	proteolysis	GO:0006508	*lap3*, *usp3*, *psmb3*, *proza*, *zmpste24*, *ctsh*, *psma8*, *pmpca*	0.003
		glycine catabolic process	GO:0006546	*amt*, *gldc*	0.007
		ribosome biogenesis	GO:0042254	*dcaf13*, *sdad1*, *nip7*	0.016
		proteolysis involved in cellular protein catabolic process	GO:0051603	*psmb3*, *ctsh*, *psmab*	0.019
		protein methylation	GO:0006479	*armt1*, *hemk1*	0.048
	cellular component	nucleus	GO:0005634	*cmpk*, *egr1*, *prpf31*, *sdad1*, *dusp22a*	0.039
	molecular function	hydrolase activity	GO:0016787	*enpp6*, *dusp22a*, *ddx56*, *hdac3*, *usp3*, *psmb3*, *smpd1*, *ovca2*, *ctsh*, *psma8*	0.006
		peptidase activity	GO:0008233	*usp3*, *psmb3*, *zmpste24*, *ctsh*, *psma8*	0.030
	KEGG pathway	Biosynthesis of antibiotics	dre01130	*dbt*, *aldh7a1*, *amt*, *zmpste24*, *psat1*, *mdh2*, *gldc*	0.0008
		Glycine, serine and threonine metabolism	dre00260	*aldh7a1*, *amt*, *psat1*, *gldc*	0.001
		Glyoxylate and dicarboxylate metabolism	dre00630	*amt*, *mdh2*, *gldc*	0.011
		Metabolic pathways	dre01100	*cmpk*, *lap3*, *dbt*, *aldh7a1*, *amt*, *smpd1*, *atp6ap1a*, *zgc*:*92907*, *coq7*, *psat1*, *ndufs2*, *mdh2*, *gldc*	0.012
		Carbon metabolism	dre01200	*amt*, *past1*, *mdh2*, *gldc*	0.023
PSGs	biological process	phospholipid biosynthetic process	GO:0008654	*chpt1*, *tamm41*	0.038
	cellular component	mitochondrion	GO:0005739	*ndufb8*, *hoga1*, *echdc3*, *mrrf*, *tamm41*	0.018
		mitochondrial respiratory chain complex I	GO:0005747	*ndufb8*, *ndufa11*	0.041

## Discussion

Given that yellow perch populations are widely distributed in the North American continent, and isolated by both geographic and genetic distances to some extent [66], we could imply that the southeast populations had already adapted to the warmer regions. The yellow and European perch diverged at 19.8 million years ago during the early Miocene Epoch, while the divergence time among *Perca*, pike perch, and ruffe should also be tens of million years ago [60]. Thus, environmental stress should have accumulated enough selection during the adaptive evolution among these species. In the respect to evolution, high ratios of d_N_/d_S_ generally suggest the frequent occurrence of adaptive evolution with a high rate of functional protein divergence arising from direction selection, which also indicates the role and strength of natural selection in phenotypic evolution and divergence among species [67]. With comparisons among genomes, one can study the evolution of genes and other genomic sequences and how molecular evolution relates to adaptation and phenotypic evolution at the organismic level, concerning fast-evolving and positively selected genes that attribute to natural selection on beneficial alleles in driving DNA sequence evolution.

In this study, we used assembled transcriptomes from RNA-seq to explore fast-evolving genes and positively selected genes involved in evolutionary mechanisms that potentially support ecological adaptation of temperature and body shape among these four Percid species. Recent studies have provided references and evidence for the role and mechanisms of such kind of genes in adaptation. For examples, similar researches were performed on cactophilic *Drosophila*, and the Tibetan fish *Gymnodiptychus pachycheilus* to identify potential candidate genes for environmental adaptation [68, 69]. Similarly, comparative transcriptome analysis in alvinellid polychaetes revealed that the trait of thermophilic species that still inhabit higher temperature environments was maintained by purifying selection in lineages, while the trait of lineages currently living in colder habitats was likely obtained under selective relaxation, with some degree of positive selection for low-temperature adaptation at the protein level [70].

### Evolutionary differences between cold and warm adaptation

After screening the 1,027 one-to-one orthologous genes in the software FasParser2, there were 22 FEGs with high d_N_/d_S_ ratios shared among the three cold-tolerant sympatric species, *P*. *fluviatilis*, *G*. *cernua*, and *S*. *lucioperca*. These 22 FEGs were significantly enriched in “translational initiation” with two eukaryotic initiation factors (eIFs), *eif1ad* and *eif3s6ip*. For decades, it has been reported that translation-initiated proteins synthesis always target some vital functions during cold adaptation, including: 1) eliminating unnecessary secondary structures of nucleic acids at low temperature [71, 72]; 2) balancing the membrane fluidity to resist cold stress [73], as membrane stiffness caused by cold can lead to the deterioration of membrane-related cell functions [74, 75]; and 3) inducing the synthesis of specific cold-protective proteins and glycoproteins, especially key cold shock proteins (CSPs) [76–79]. Additionally, the *cse1l* (alias of CAS, one of the 22 FEGs), a specific nuclear transport factor that transports importin and exportin alpha between the nucleus and cytoplasm [80], downregulated the *cftr* activity to keep fluid homeostasis [81]. Obviously, fluid secretion and homeostasis are primary strategies for fish in withstanding environmental stress, especially the epidermal mucus as the first barrier [82].

Other three fast-evolving genes (*psmd12*, *fbxo45*, and *anapc2*) were found to be shared by the three cold-related Percid species examined here. These genes are implicated in the process of protein ubiquitination, a process that seems to be relevant for cold-temperature tolerance. The role of protein ubiquitination level was demonstrated in three Antarctic fish, showing a higher level of protein ubiquitination than fish living in warmer environments at lower latitude [83]. A large number of studies showed that the protein ubiquitination process directly mediated response to cold. This is because the ubiquitin 26S proteasome system could directly regulate cold signaling and induce the ICE (inducer of CBF expression), which controlled the expression of cold-responsive transcription factor CBF3/DREB1A that regulated the transcription of numerous cold-responsive genes [84–87].

In contrast, the warm-tolerant perch *P*. *flavescens* showed, also, significant genes that seemed to have a vital role in the process of adapting to warmer temperatures. Among the 93 FEGs in yellow perch, the most significantly enriched molecular function term was “peptidyl-prolyl cis-trans isomerase activity” (*p < 0*.*001*). As reported, these isomerases increased in intermediate in the process of protein folding [88]. Notably, among the four genes (*ttc9b*, *ppil1*, *fkbp3*, *pin4*) related to “peptidyl-prolyl cis-trans isomerase activity,” *ttc9b* and *pin4* were both fast-evolving and positively selected. As one of the FK506-binding proteins, we supposed that *fkbp3* (*fkbp25*) functioned in a similar role as other members like *fkbp38*, *fkbp51*, *fkbp52*, and *fkbp54*. They associated with heat shock proteins (especially HSP70, HSP90), and had peptidyl-prolyl cis-trans isomerase activity [89–92]. Then the most significantly and positively selected biological process was the “SCF-dependent proteasomal ubiquitin-dependent protein catabolic process” (*p < 0*.*001*). The SCF (Skp, Cullin, F-box containing proteins) and SCF-like complexes regulate large numbers of protein processes involved in cell cycle progression, DNA damage response, and signal transduction and transcription [93–95]. Interestingly, most of the F-box proteins showed the tendency of high temperature induction [96], and the two F-box genes, *fbxl5* and *fbxw5*, were both fast-evolving and positively selected in *P*. *flavescens*. Likewise, the “Cul4-RING E3 ubiquitin ligase complex” was also significantly enriched and positively selected. A study on *Arabidopsis* revealed that Cullin4-RING ubiquitin ligase participates in heat stress response through its association with HSP90-1 [97]. Meanwhile, the RING-type E3 in rice promoted tolerance of heat stress via mediating re-localization of nuclear proteins [98, 99].

Besides the first two most significantly enriched terms, two CC terms, “endoplasmic reticulum membrane” and “cytoplasm,” which were significantly enriched in both FEGs and PSGs in *P*. *flavescens*, should also be paid attention. This might imply that these two fast-evolving and positively selected functional terms might be specific and important within the *P*. *flavescens* lineage for adapting to the warmer environment of the Great Lakes and farther south, as the “protein processing in endoplasmic reticulum” was proved to be a heat-specific pathway in *D*. *rerio* [63]. Under heat stress, the primary reaction of most organisms is the intervention of HSPs [100–102]. Most HSP granules are usually synthesized or located in the cytoplasm with the help of endoplasmic reticulum and subsequently translocated into the nucleus or involved in signal transduction from cytoplasm to the nucleus under stress conditions [103–105]. Similarly, the “oxidoreductase activity” and “oxidation-reduction process” should also be directly counted into the adaptation under thermal stress [106, 107].

In addition to the functional terms and genes mentioned above, the following naturally selected genes in *P*. *flavescens* might also reveal more profound adaptive information, most of which were directly assigned to energy or lipid metabolism. For instance, the fast-evolving gene *cnep1r1* and positively selected gene *mgll* concern lipid metabolic process. Most fish, when exposed to environmental stimuli, typically require more energy consumption, and lipids are one kind of main energy stores for fish [108]. The long-term temperature acclimation research on zebrafish showed that lipid and liver protein consumptions were increased when exposed to high temperature [109], which could provide extra evidence that lipids play key roles in heat response management [110]. What is more, phosphorylation, the basic activator of heat-induced genes [110–112], was revealed by three warm-related FEGs (*phlda3*, *gtf2h4*, *stk10*) rather than those cold-related FEGs mentioned above.

Altogether, we could hypothesize the evolutionary differences between one cultured and three wild Percidae species for adapting to divergent temperature environments, which, of course, are on the basis of fundamental processes like energy metabolism, signal transduction, and membrane and cell proliferation/apoptosis [113, 114].

#### Cold-adaptation

Since *P*. *fluviatilis*, *G*. *cernua*, and *S*. *lucioperca* originated from and are mainly distributed in the northwest of Eurasia, responses to the cold should be the most basic physiological mechanism. Cold might stimulate and accelerate their translation-initiated proteins synthesis, so that they could store enough cold-protective proteins and glycoproteins, especially CSPs. Genes like *eIFs*, *cse1l*, *psmd12*, *fbxo45*, and *anapc2* present fast evolutionary rates or are naturally selected during the long term of cold adaptation, and they subsequently undertake their respective molecular functions or biological processes for responding to environmental stress. Some of these genes could balance membrane fluidity and secrete epidermal mucus to resist the cold stress. The induced protein ubiquitination process could, in return, regulate the cold signaling and mediate the transcription of numerous cold-responsive genes.

#### Warm-adaptation

The key point should be the interaction of HSPs and the chaperones. At this point, *ttc9b*, *ppil1*, *rnf7*, *fbxw5*, *fkbp3*, and *pin4* are significantly involved and naturally selected in *P*. *flavescens*, especially positive selection on synthesis of HSPs through the endoplasmic reticulum membrane in cytoplasm. Meanwhile, some auxiliary biological processes are conducted by their respective genes. First of all, F-box genes, *fbxl5* and *fbxw5*, could positively respond to heat stress. Then, *cnep1r1* and *mgll* could fuel the lipid metabolic process to compensate the acute energy consumption under heat stress, as well as play key roles in heat response management for *P*. *flavescens*. Not surprisingly, *phlda3*, *gtf2h4*, and *stk10* might also urge the phosphorylation to activate heat-induced genes. Gradually, *rnf7* and *fbxw5* are positively selected by nature so that they could better exercise the power of “Cul4-RING E3 ubiquitin ligase complex,” promoting the tolerance of heat stress for *P*. *flavescens*. Eventually, these evolutionary issues in *P*. *flavescens* should contribute to its adaptation to the warmer Great Lakes and farther south during long-term evolution.

### Evolutionary differences in body shape

The shared FEGs and PSGs among three small perches (*P*. *flavescens*, *P*. *fluviatilis*, and *G*. *cernua*) were mainly involved in some vital, but basic, terms and showed little information directly related to growth. Since *P*. *flavescens* and *P*. *fluviatilis* are still very similar in biology, and body shape to *G*. *cernua*, we might hypothesize that genes related to this trait would be under purifying selection. However, the FEGs and PSGs in the large and elongated *S*. *lucioperca* implied meaningful clues for evolutionary adaptation in body shape. There were as many as 22 FEGs and 7 PSGs related to energy metabolism such as “hydrolase activity,” “mitochondrion,” “glyoxylate and dicarboxylate metabolism,” “metabolic pathways,” and “carbon metabolism.” Deeping into the 14 GO terms and KEGG pathways that significantly enriched from the FEGs and PSGs in *S*. *lucioperca*, we paid more attention to the genes that might contribute to ecological adaptation in body shape.

To begin with, the *aldh7a1*, with the oxidoreductase activity of aldehyde dehydrogenase (aldh) family members that are essential for eye development, plays a critical role in eye and limb development in fish, specifically in the development of camera-type eyes, cartilage, bone, and pectoral fin [115–117]. Together, the *egr1* [118–121] and *prpf31* [122, 123] are also necessary for retina development in the camera-type eye. Whilst *egr1* is also a transcriptional regulator of numerous target genes, it thereby plays a crucial role in regulating the response to growth factors [124]. Similarly, the *hdac3*, a member of histone deacetylases (HDACs) that is involved in multiple developmental processes [125, 126], plays a key role in the regulation of posterior lateral line formation and provides evidence for epigenetic regulation in auditory organ development [127].

The *sdad1* was assigned to a significant biological process called “actin cytoskeleton organization.” As it is known, actin participates in many important cellular processes including muscle contraction, cell division and cytokinesis, cell signaling, and the establishment and maintenance of cell junctions and cell shape [128–130]. In vertebrates, muscle tropomyosin, isoforms of actin found in muscle tissues, is a major constituent of muscle contractile apparatus [131, 132]. Likewise, the alpha-actin was proved to be essential for the development of cardiac and skeletal muscles [133–135]. Notably, skeletal muscles are the force-generators of the body [136–138], the strength of which largely determines the swimming speed of fish [139–141]. However, the apparent role of ubiquitin/proteasome pathway in skeletal muscle growth should not be ignored, as proper synthesis and degradation of the appropriate myogenic proteins is indispensable during myogenic process [138, 142, 143]. In this study, the two fast-evolving genes *psmb3* and *psma8* (proteasome subunit beta/alpha type) were assigned to “proteasome-mediated ubiquitin-dependent protein catabolic process.”

Last but not least, the two fast-evolving genes *amt* and *gldc* were involved in “glycine catabolic process.” In the 1980s, uptake of glycine by fish scales indicated protein synthetic compensation under cold exposure, and was regarded as an index of fish growth [144, 145]. In particular, glycine uptake by scales of colder acclimated fish showed a higher rate for a given growth rate [146]. More importantly, glycine, one kind of high energy storage in fish [147], participates in gluconeogenesis, carbon metabolism, fat digestion [148], stimulating feed intake [149], regulation of osmoregulatory responses [150], and may also regulate gene expression in rainbow trout [151]. More powerful evidence of glycine relating to growth was found in the study on the effect of glycine supplementation on growth performance, body composition, and salinity stress of juvenile Pacific white shrimp, which highlighted that supplementation of glycine can increase weight gain, affect the amino acid content of muscle, and increase the anti-oxidative capacity of white shrimp [152].

Based on findings from this study, we could propose the following potential evolutionary advantages driven by genes that are influenced by natural selection, for the elongated body shape of *S*. *lucioperca*. In addition to some basic metabolic mechanisms established by a large amount of genes, genes like *aldh7a1*, *egr1*, and *prpf31* ensure the development of the camera-type eye, which, together with posterior lateral line, could help *S*. *lucioperca* better sense the surrounding environment and lock onto prey [153, 154]. Thus, it could predict danger and ensure high predation efficiency. With the help of *amt* and *gldc*, its appetite increases and energy intake is sufficient, guarantying a high growth rate. In the meantime, the absorption of glycine by scales could help to establish an effective surface barrier. Then the *hdac3*, together with *aldh7a1*, could shape the body to be elongated and develop an effective pectoral fin, which is more conducive to shuttle in the rushing, complex water [155–158]. Meanwhile, *sdad1*, *psmb3*, and *psma8* work closely together to build muscle strength so that *S*. *lucioperca* can swim faster and effectively catch prey. More or less, these natural selected molecular responses and related physiological traits should contribute to its evolution to its high position on the food web and adapting to this important ecological niche.

## Conclusions

The four Percidae fish involved in this study showed differences in adaptation to temperature environment and body shape, to some extent. Although the findings of this study are not able to confirm any "signature of selection", there are indications that selective processes in the transcriptome could be enacted to allow these Percidae fish to locally adapt to different ranges of temperature, and explain the evolutionary difference in body shape, to some extent. We identified the fast-evolving and positively selected genes among these four Percidae fish with *O*. *niloticus* and *D*. *rerio* as references, so as to predict molecular insights into ecological niche partitioning and divergent adaptation involved in the evolutionary race [159, 160]. However, referring to Stepien et al.’s comprehensive study on evolutionary and adaptive issues of perch [66], we realized that our study lacked sufficient geographic populations and phylogenetic species to emphasize more powerful and inherent mechanisms responsible for evolution. Moreover, quantitative trait locus (QTL) were also necessary for expounding the power of natural selection and genetic mutation/drift [161]. Nevertheless, the naturally selected genes and mechanisms presented in this study attract our further interest in studying the influence of temperature on the adaptation and growth of fish.

## Supporting information

S1 Table Water temperature of Lake Erie and ponds in the OSU South Centers. (XLSX)

S2 Table Monitored water temperature in the Irtysh River during warmer seasons. (XLSX)

S3 Table Basic morphological data for the four species involved. (XLSX)

S4 Table dN/dS ratios revealed from 1,027 orthologues among the six species. (XLSX)
